# Identification of Rare Copy Number Variants Associated With Pulmonary Atresia With Ventricular Septal Defect

**DOI:** 10.3389/fgene.2019.00015

**Published:** 2019-01-28

**Authors:** Huilin Xie, Nanchao Hong, Erge Zhang, Fen Li, Kun Sun, Yu Yu

**Affiliations:** ^1^Department of Pediatric Cardiology, Xin Hua Hospital, School of Medicine, Shanghai Jiao Tong University, Shanghai, China; ^2^Department of Pediatric Cardiology, Shanghai Children’s Medical Center, School of Medicine, Shanghai Jiao Tong University, Shanghai, China

**Keywords:** copy number variants, congenital heart defects, pulmonary atresia with ventricular septal defect, whole exome sequencing, network, PPP4C, FLT4, RICTOR

## Abstract

Copy number variants (CNVs) are major variations contributing to the gene heterogeneity of congenital heart diseases (CHD). pulmonary atresia with ventricular septal defect (PA-VSD) is a rare form of cyanotic CHD characterized by complex manifestations and the genetic determinants underlying PA-VSD are still largely unknown. We investigated rare CNVs in a recruited cohort of 100 unrelated patients with PA-VSD, PA-IVS, or TOF and a population-matched control cohort of 100 healthy children using whole-exome sequencing. Comparing rare CNVs in PA-VSD cases and that in PA-IVS or TOF positive controls, we observed twenty-two rare CNVs only in PA-VSD, five rare CNVs only in PA-VSD and TOF as well as thirteen rare CNVs only in PA-VSD and PA-IVS. Six of these CNVs were considered pathogenic or potentially pathogenic to PA-VSD: 16p11.2 del (PPP4C and TBX6), 5q35.3 del (FLT4), 5p13.1 del (RICTOR), 6p21.33 dup (TNXB), 7p15.2 del (HNRNPA2B1), and 19p13.3 dup (FGF22). The gene networks showed that four putative candidate genes for PA-VSD, PPP4C, FLT4, RICTOR, and FGF22 had strong interaction with well-known cardiac genes relevant to heart or blood vessel development. Meanwhile, the analysis of transcriptome array revealed that PPP4C and RICTOR were also significantly expressed in human embryonic heart. In conclusion, three rare novel CNVs were identified only in PA-VSD: 16p11.2 del (PPP4C), 5q35.3 del (FLT4) and 5p13.1 del (RICTOR), implicating novel candidate genes of interest for PA-VSD. Our study provided new insights into understanding for the pathogenesis of PA-VSD and helped elucidate critical genes for PA-VSD.

## Introduction

Pulmonary atresia with ventricular septal defect (PA-VSD) is a kind of rare complex manifestations of congenital heart diseases (CHD), characterized by the lack of luminal continuity and blood flow from either the right ventricle and the pulmonary artery, together with ventricular septal defect (Digilio et al., 1996; Tchervenkov and Roy, 2000). PA-VSD is considered as one of the most complex and unmanageable CHD, with an estimated prevalence of 0.2% of live births and roughly 2% in congenital heart defects (Hoffman and Kaplan, 2002; Abid et al., 2014). Surgical interventions and medical care are always needed for patients with PA-VSD, nevertheless, PA-VSD remains a leading cause of neonatal death (Leonard et al., 2000; Amark et al., 2006).

Copy number variants (CNVs) contribute to the gene heterogeneity of CHD (Soemedi et al., 2012; Tomita-Mitchell et al., 2012; Warburton et al., 2014), providing important genetic information of complex CHD. Previous studies showed the 22q11.2 deletion was a well-known pathogenic variant in CHD and was most common in tetralogy of Fallot (TOF) and PA-VSD (Momma, 2010; Xu et al., 2011; Warburton et al., 2014). Deletions in 15q11.2 and 8p23.1 also contribute to the risk of sporadic CHD (Soemedi et al., 2012). Additionally, some rare CNVs and relevant genes were associated with pulmonary atresia (PA-IVS and PA-VSD), such as 5q14.1dup (DHFR), 10p13dup (CUBN), and 17p13.2del (CAMTA2) (Xie et al., 2014). However, it lacks genetic evidence of PA-VSD in current studies and the majority of them have typically focused on the diagnostic instruments and surgical procedures; the genetic determinants underlying PA-VSD are still needed to be identified.

The aim of our study is to determine the contribution of rare CNVs in the etiology of sporadic PA-VSD and distinguish the genetic pattern between PA-VSD and PA-IVS or TOF. Here we genotyped sixty PA-VSD patients with the whole-exome sequencing and investigated the same or different rare CNVs in PA-VSD compared to non-PAVSD CHD cohort (PA-IVS or TOF) to explain their common or diverse phenotypes. In addition, we detected putative candidate genes encompassed in rare CNVs and identified functional gene sets associated with heart development through gene network analysis.

## Materials and Methods

### Study Population

We recruited unrelated patients with PA-VSD (*n* = 60) or TOF (*n* = 20) or PA-IVS (*n* = 20), diagnosed by echocardiogram, cardiac catheterization, or surgery from Shanghai Xin Hua Hospital. Patients with TOF and PA-IVS were as a non-PAVSD CHD cohort and 100 healthy children without heart diseases were as controls. Written informed consents were obtained from the parents or guardians of participants in this study. The study was conducted in accordance with the Declaration of Helsinki, and the protocol was approved by the Ethics Committee of Xin Hua Hospital. The genomic DNA of participants was extracted by using the QIAamp DNA Blood Mini Kit (QIAGEN, Germany) following the manufacturer’s instructions and was then stored at -80 °C.

### Whole-Exome Sequencing and Data Analysis

The whole exome sequencing was performed for copy number variations (CNVs) in all participants. Whole exome sequencing data sequenced by HiseqTM Sequencer was filtered (removing the adaptor sequences, reads with >5% ambiguous bases (noted as N) and low-quality reads containing more than 20 percent of bases with qualities of <20) and mapped to Cattle genome (Human genome Version GRCh37 Ensembl75 NCBI) utilizing BWA-mem under following parameter (bwa mem -t 8 -R) (Li and Durbin, 2010). Duplicated reads were marker by PICARD^1^ and recalibration was applied based on the GATK standard calling pipeline tools^2^.

### CNV Determination From WES Data

CNVkit (Talevich et al., 2016) was used to calculate the CNV in the WES analysis. This method applies the copy number in control group as base line and CNVs below 1.5 times than baseline in case group were excluded. Then CNVs were analyzed with the Database of Genomic Variants (DGV^3^) and the overlapped CNV region was filtered. CNVs were excluded if they were shorter than 10 kb.

### Tissue Collection and Transcriptome Array

Human embryos from Carnegie stages 10–16 were acquired after medical termination of pregnancy at Shanghai Xin Hua Hospital. The medical ethics committee of Xin Hua Hospital approved the study. Human embryonic heart samples were remained for transcriptome array. TissueLyserII (Qiagen) and the RNeasy MinElute Cleanup Kit (Qiagen) were utilized for RNA extraction. The integrity and purity of the RNA was detected by the Experion automated gel electrophoresis system (Bio-Rad) and the NanoDrop 2000c spectrophotometer (ThermoFisher Scientific). The time course expression patterns of the candidate genes were measured using an Affymetrix HTA 2.0 microarray.

### Network Analysis

We used the bioinformatic software, Cytoscape, with STRING database to perform network analysis. Three different gene lists derived from previous literatures and MalaCards database^4^ were used. The lists were constructed as follows: (1) Genes associated with CHD outflow tract development, the secondary heart field (SHF) or cardiac neural crest (CNC) from previous studies; (2) Genes involved in blood vessel development; (3) Genes related to well-known syndromes with heart defects from previously reported studies and database (Supplementary Table S1). We selected 30 genes from the rare CNV loci and then severally analyzed the network between these genes and the three gene lists.

## Results

### Identification of Rare CNVs

Of the 100 patients, sixty were PA-VSD, twenty were TOF and another twenty were PA-IVS. The patients are from the Chinese Han population with ages ranging from 2 months to 13 years (Table 1). We studied the 100 patients genotyped by WES analysis.

**Table 1:** Cardiac diagnoses for study population of patients.

Diagnoses	Number	Gender/Number	Age
PA-VSD	60	F21 M39	2m-13y
PA-IVS	20	F10 M10	2m-10y
TOF	20	F8 M12	2m-2y
total	100	F39 M61	2m-13y


*PA-VSD, pulmonary atresia with ventricular septal defect; PA-IVS, pulmonary atresia with intact ventricular septum; TOF, tetralogy of Fallot; F, female; M, male; d, day(s); m, month(s); and y, year(s).*

**FIGURE 1:**
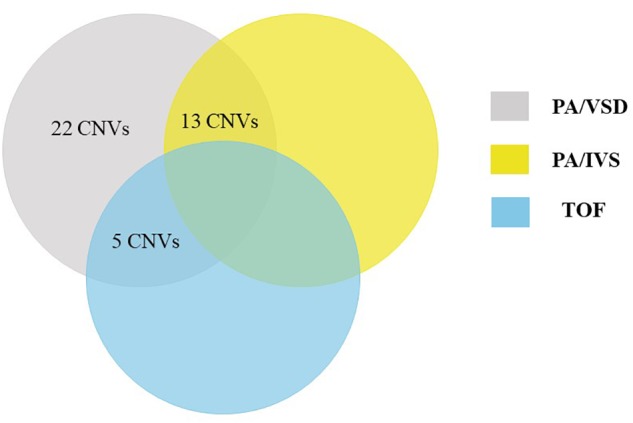
Venn diagram outlining overlap between rare CNVs in PA-VSD (gray), PA-IVS (yellow), and TOF (blue). We compared rare CNVs in PA-VSD cases with that in PA-IVS or TOF positive controls. There were twenty-two rare CNVs only in PA-VSD, five rare CNVs only in PA-VSD and TOF as well as thirteen rare CNVs only in PA-VSD and PA-IVS.

Using a stringent CNV analysis strategy described in the Methods, 129 CNVs were identified and 66 (51%) were duplications and 63 (49%) were deletions. These CNVs had been analyzed with DGV for overlap, which not detected in the DGV were considered as rare CNVs. Moreover, rare CNVs were excluded if they were shorter than 10 kb. We compared rare CNVs in PA-VSD cases and PA-IVS or TOF positive controls and there were twenty-two rare CNVs only in PA-VSD, five rare CNVs only in PA-VSD and TOF as well as thirteen rare CNVs only in PA-VSD and PA-IVS (Figure 1).

### Rare CNVs Only in PA-VSD

Twenty-two rare CNVs were only identified in PA-VSD with a size range from 12.3 to 344.5 kb (Table 2). Among these rare CNVs, some have been reported to implicated in CHD. The most compelling was 16p11.2 deletion previously detected in a neonate with TOF with pulmonary atresia (Hernando et al., 2002) and identified in a CHD cohort (Zhu et al., 2016). Besides, the duplication of 1q21.2 and 17q12 were previously related to TOF (Liu et al., 2016). In a previous study, the 13q33.3 deletion, together with a 4p12 duplication were detected in a patient with double outlet right ventricle (McMahon et al., 2015). However, the deletion of 4p12 and 13q33.1 (near 13q33.3 locus) were observed in the same patient in our study. Additionally, there were two rare CNVs previously relevant to syndromes with heart defects: one was 11q23.3 deletion involving Jacobsen syndrome with severe cardiac malformations (Mattina et al., 2009), and another was 5q35.3 deletion correlated with 5q35.3 subtelomeric deletion syndrome which showed developmental delay and CHD (Rauch et al., 2003).

**Table 2:** Rare CNVs only in PA-VSD.

Locus	Start	End	Size (bp)	CN	Genes	Cases
6p21.33	31779686	31797413	17727	gain	HSPA1L, HSPA1A, HSPA1B	PA_VSD111, PA_VSD21, PA_VSD22, PA_VSD27, PA_VSD37
15q15.3	43886386	43910353	23967	gain	CKMT1B	PA_VSD21, PA_VSD58, PA_VSD38
22q13.2	42522572	42538657	16085	gain	CYP2D6	PA_VSD110, PA_VSD27, PA_VSD151
10p12.33	17875713	17952468	76755	loss	LOC101928757, MIR511	PA_VSD116, PA_VSD35
5p13.1	38924520	38965026	40506	loss	OSMR, RICTOR	PA_VSD14, PA_VSD29
9q34.12	131009642	131038507	28865	gain	SWI5	PA_VSD107, PA_VSD46
1q21.2	149815116	149832746	17630	gain	HIST2H2BD, HIST2H2BC, HIST2H2AA4, HIST2H3A, HIST2H4B	PA_VSD37, PA_VSD46
16p11.2	29465517	29478590	13073	gain	SLX1B, SLX1B-SULT1A4, SULT1A4, LOC388242	PA_VSD13, PA_VSD5
16p11.2	29790460	30134962	344502	loss	ZG16, KIF22, MAZ, LOC100289283, PRRT2, PAGR1, MVP, CDIPT, CDIPT-AS1, SEZ6L2, ASPHD1, KCTD13, TMEM219, TAOK2, HIRIP3, INO80E, DOC2A, C16orf92, FAM57B, ALDOA, PPP4C, TBX6, YPEL3, LOC101928595, GDPD3, MAPK3	PA_VSD130
11q23.3	118037598	118134881	97283	loss	SCN2B, AMICA1, MPZL3, MPZL2	PA_VSD116
5q35.3	179991469	180042160	50691	loss	SCGB3A1, FLT4	PA_VSD111
4p12	44682653	44724286	41633	loss	GUF1, GNPDA2	PA_VSD130
6p22.1	27775621	27806847	31226	gain	HIST1H2BL, HIST1H2AI, HIST1H3H, HIST1H2AJ, HIST1H2BM, HIST1H4J, LOC100996513, HIST1H4K, HIST1H2BN, HIST1H2AK	PA_VSD37
13q33.1	103381765	103411283	29518	loss	CCDC168	PA_VSD130
11p15.5	1003620	1031082	27462	gain	AP2A2, LOC101927462, MUC6	PA_VSD38
2p16.1	58366794	58392993	26199	loss	VRK2, FANCL	PA_VSD130
18p11.21	14828224	14852418	24194	loss	ANKRD30B, MIR3156-2	PA_VSD42
15q15.3	43986218	44009815	23597	gain	CKMT1A	PA_VSD19
11q12.1	60164032	60184537	20505	loss	MS4A14	PA_VSD107
10q23.31	90350323	90366691	16368	loss	LIPJ	PA_VSD130
17q12	39182910	39197477	14567	gain	KRTAP1-5, KRTAP1-4, KRTAP1-3, KRTAP1-1	PA_VSD138
2p23.2	27789783	27802104	12321	loss	LOC100420668, C2orf16	PA_VSD130


*PA-VSD, pulmonary atresia with ventricular septal defect; Locus, cytogenetic location of CNV; Size, in base pairs; CN, type of copy number aberration.*

**FIGURE 2:**
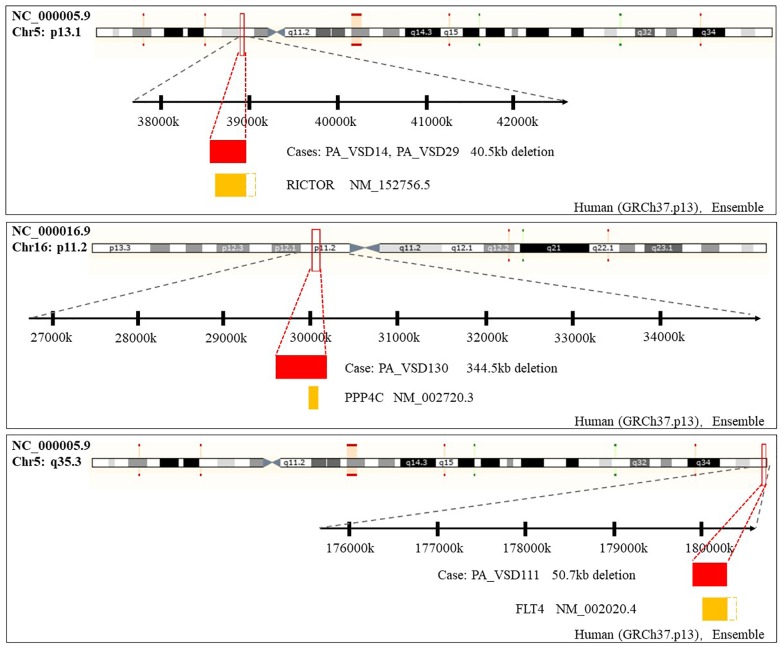
Rare CNVs overlapping novel candidate gene for PA-VSD: RICTOR, PPP4C, and FLT4. The dotted rectangles represent the part of candidate genes which are not within the CNVs. Genomic parameters from Ensembl (GRCh37.p13).

Amongst these rare CNVs of note, the deletions of 16p11.2 and 5q35.3 implicated specific candidate genes of interest. The 344.5 kb 16p11.2 deletion included two candidate genes: PPP4C (BMP signaling pathways) and TBX6 (T-box family), and we considered that they might have an impact on cardiac development or are implicated by a relevant family of genes. We also identified a 50.7 kb deletion at 5q35.3 locus containing the FLT4 gene, which are also called VEGFR3 (Ferrara and Alitalo, 1999). Another interesting rare CNV identified in two patients with PA-VSD was 5p13.1 deletion containing the RICTOR gene which played a crucial role in heart development (Figure 2).

### Rare CNVs in PA-VSD and TOF

Pulmonary atresia with ventricular septal defect shows similar phenotype and hemodynamics to TOF with PA, so sometimes PA-VSD is considered as the most severe type of TOF. To explain the possible similar development mechanism of PA-VSD and TOF, we compared the rare CNVs in PA-VSD and TOF, then identified five rare CNVs in both PA-VSD and TOF and they were 6p21.33 duplication, 22q13.2 duplication, 7p22.1 deletion, 16q22.1 duplication, and 15q21.1 deletion (Table 3). A duplication of 15q21.1 (chr15: 48023616-49017024) was previously reported to associate with CHD and identified in a patient with TOF and PA (Molck et al., 2017). However, the deletion of 15q21.1 spanned approximately 17.8 kb in our study did not include the vital genes involving in heart morphogenesis.

**Table 3:** Rare CNVs only in PA-VSD and TOF.

Locus	Start	End	Size (bp)	CN	Genes	Cases
6p21.33	32016526	32036947	20421	gain	TNXB	PA_VSD111, PA_VSD115, PA_VSD21, PA_VSD22, PA_VSD27, PA_VSD37, PA_VSD51, PA_VSD58, TOF129, TOF148
22q13.2	42897617	42915769	18152	gain	SERHL, LOC101927372, RRP7A	PA_VSD24, PA_VSD38, PA_VSD39, PA_VSD110, PA_VSD113, TOF124
7p22.1	6785685	6864382	78697	loss	PMS2CL	PA_VSD135, PA_VSD53, TOF121
16q22.1	70161181	70190826	29645	gain	PDPR	PA_VSD39, TOF149
15q21.1	48443249	48461040	17791	loss	MYEF2	PA_VSD14, TOF125


*PA-VSD, pulmonary atresia with ventricular septal defect; TOF, tetralogy of Fallot; Locus, cytogenetic location of CNV; Size, in base pairs; CN, type of copy number aberration.*

An interesting CNV in eight PA-VSD patients and two TOF patients at the 6p21.33 locus was observed as a recurrent rare event, and the gain CNV overlapped the TNXB gene, which was reported to be highly expressed in fetuses and pregnancies with isolated ventricular septal defects (VSD) (Arcelli et al., 2010; Morano et al., 2018).

### Rare CNVs in PA-VSD and PA-IVS

Although the genetic developmental patterns of PA-VSD are partly different from that of PA-IVS, we believe that the common CNVs and genes in these two populations may help explain the similarity in phenotypes. In this study, PA-VSD and PA-IVS had thirteen rare CNVs identified in common (Table 4). We identified a duplication of 2q37.1 in four PA-VSD patients and one PA-IVS patients. It was previously reported that 2q37 microdeletion syndrome showed developmental delay and congenital heart disease phenotypes (Doherty and Lacbawan, 2007).

**Table 4:** Rare CNVs only in PA-VSD and PA-IVS.

Locus	Start	End	Size (bp)	CN	Genes	Cases
19p13.2	8986992	9091771	104779	gain	MUC16	PA_VSD32, PA_VSD49, PA_VSD4, PA_VSD27, PA_VSD40, PA_VSD45, PA_IVS63
2q32.1	186610162	186697940	87778	loss	FSIP2, LOC100420895	PA_VSD112, PA_VSD130, PA_VSD134, PA_VSD29, PA_IVS62
11q14.3	89370649	89451020	80371	loss	TRIM77	PA_VSD101, PA_VSD112l, PA_VSD130, PA_VSD55, PA_IVS120
2q37.1	233243677	233274613	30936	gain	ALPP, ECEL1P2, ALPPL2	PA_VSD113, PA_VSD136, PA_VSD53, PA_VSD42, PA_IVS119
15q15.3	43923716	43975639	51923	loss	STRC, CATSPER2, PPIP5K1P1	PA_VSD112, PA_VSD112, PA_IVS61
19p13.3	603581	649792	46211	gain	POLRMT, FGF22, RNF126	PA_VSD27, PA_VSD43, PA_IVS71
12q21.31	85408233	85450970	42737	loss	TSPAN19, LRRIQ1	PA_VSD29, PA_IVS143
6q24.3	146240456	146276155	35699	loss	SHPRH, LOC101928598	PA_VSD130, PA_VSD29, PA_IVS67
13q13.3	35730170	35758241	28071	loss	NBEA	PA_VSD14, PA_IVS143
16p11.2	28606954	28631399	24445	gain	SULT1A2, SULT1A1, LOC101929366	PA_VSD112, PA_VSD53, PA_IVS67
7p15.2	26222835	26236674	13839	loss	HNRNPA2B1	PA_IVS61, PA_VSD134
7q22.1	99817552	99831375	13823	gain	GATS, PVRIG	PA_VSD136, PA_IVS80
5q13.2	70234620	70248318	13698	loss	SMN1	PA_IVS142, PA_VSD112


*PA-VSD, pulmonary atresia with ventricular septal defect; PA-IVS, pulmonary atresia with intact ventricular septum; Locus, cytogenetic location of CNV; Size, in base pairs; CN, type of copy number aberration.*

In this group, we focused on 7p15.2 deletion and 19p13.3 duplication which encompassed candidate genes. The loss CNV at 7p15.2 containing the HNRNPA2B1 gene was identified in one PA-VSD patients and one PA-IVS patients. Another rare CNV at 19p13.3 locus identified in two PA-VSD patients and one PA-IVS patients contained the candidate gene FGF22.

### Expression Pattern of Candidate Genes in Human Embryonic Heart

**FIGURE 3:**
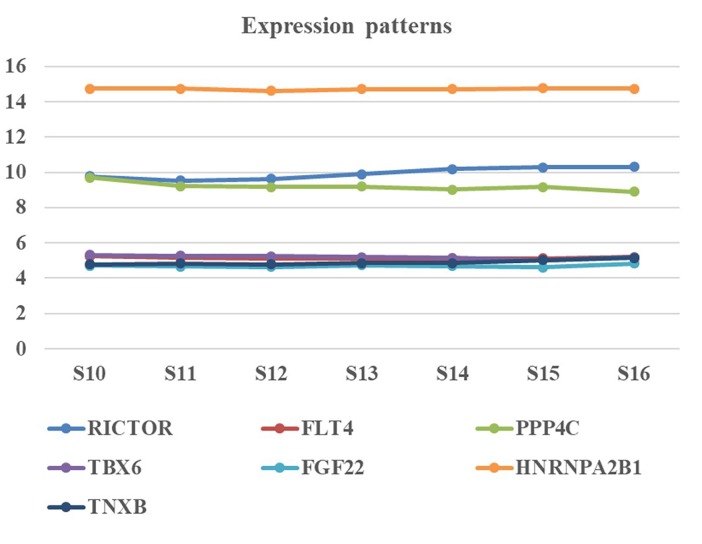
Expression pattern of candidate genes in human embryonic heart. Human embryonic heart in different Carnegie stages from S10 to S16 were performed the gene expression analysis using microarray.

We collected human embryonic heart in different Carnegie stages from S10 to S16 and performed the gene expression analysis using transcriptome array. Among these candidate genes, HNRNPA2B1 was the most highly expressed in embryonic heart; additionally, the expression levels of RICTOR and PPP4C were also significantly higher than those of other genes (Figure 3).

### Gene Networks

**FIGURE 4:**
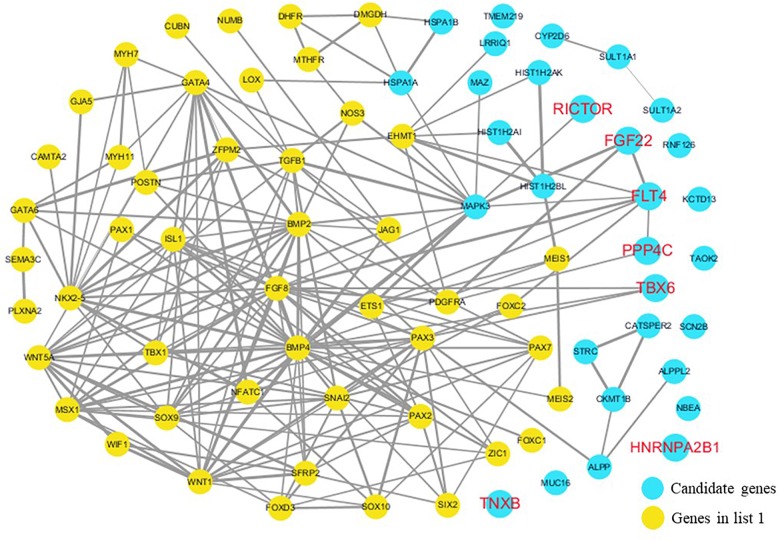
Network analysis between candidate genes and genes associated with CHD, outflow tract development, the secondary heart field (SHF) or cardiac neural crest (CNC). We used the Cytoscape, a bioinformatic software with STRING database, to perform network interaction of proteins. The red bold fonts represent candidate genes, the blue nodes represent rare CNVs loci genes in this study and the yellow nodes represent the genes in list 1. The different width of line connecting proteins represents different intensity of the protein interaction, and the wider the connecting line is, the closer the interaction is.

**FIGURE 5:**
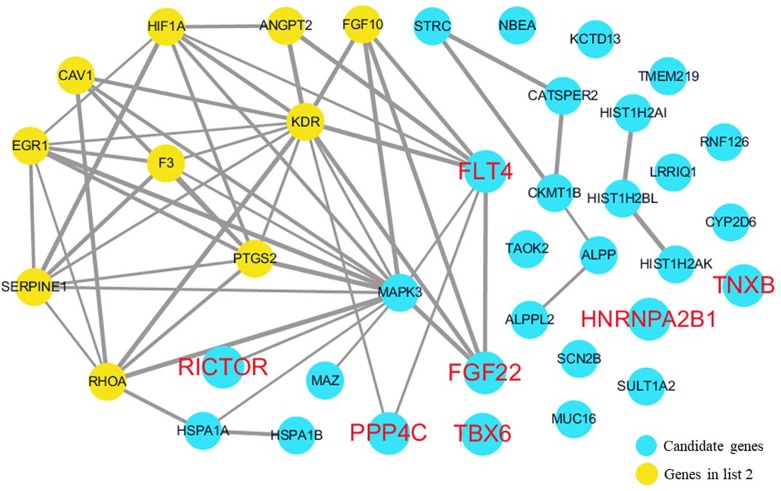
Network analysis between candidate genes and genes involved in blood vessel development. We used the Cytoscape, a bioinformatic software with STRING database, to perform network interaction of proteins. The red bold fonts represent candidate genes, the blue nodes represent rare CNVs loci genes in this study and the yellow nodes represent the genes in list 2. The different width of line connecting proteins represents different intensity of the protein interaction and the wider the connecting line is, the closer the interaction is.

**FIGURE 6:**
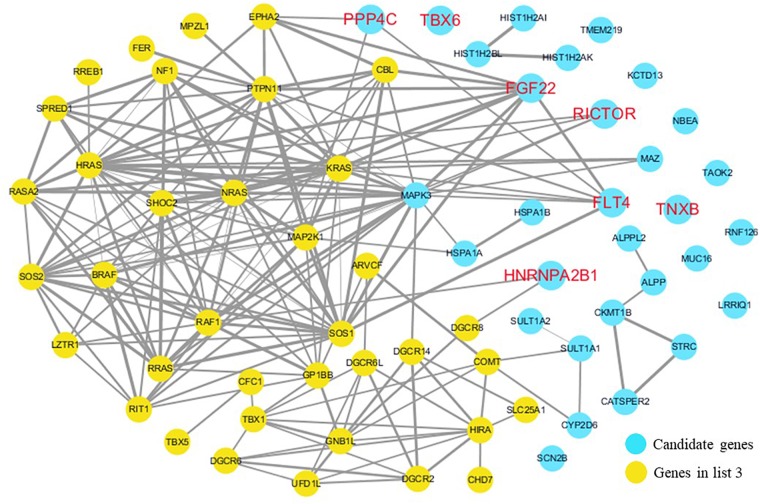
Network analysis between candidate genes and genes related to well-known syndromes with heart defects. We used the Cytoscape, a bioinformatic software with STRING database, to perform network interaction of proteins. The red bold fonts represent candidate genes, the blue nodes represent rare CNVs loci genes in this study and the yellow nodes represent the genes in list 3. The different width of line connecting proteins represents different intensity of the protein interaction, and the wider the connecting line is, the closer the interaction is.

The cardiovascular malformations of PA-VSD are caused by heart and vessel abnormally development, such as the formation and development of the cardiac outflow tract, pulmonary artery, SHF, or CNC. Additionally, multiple systemic syndromes show heart defects, like LEOPARD syndrome, Noonan syndrome, Digeorge syndrome and so on. Therefore, we consider that the genes implicated in these above aspects may play roles in the pathogenesis of PA-VSD. To detect which aspects of heart development the genes in rare CNVs identified in our study were related to, we screened previous studies and MalaCards database to get known genes about heart morphogenesis, blood vessel development and syndromes involved in heart defects, and then analyzed the networks between these candidate genes and three gene groups, respectively (Figure 4–6). We found that PPP4C, FLT4, RICTOR, and FGF22 were directly relevant to all three gene groups. TBX6 directly interacts with FGF8, BMP4 and PAX3 in gene list 1 which were related to heart development. HNRNPA2B1 directly interacts with RAF1 and DGCR8 in gene list 3 associated with syndrome. These data suggested that the four genes, PPP4C, FLT4, RICTOR and FGF22, had strong roles in cardiac development and pathogenesis of PA-VSD.

## Discussion

Copy number changes appear to be important genetic variants contributing to the etiology of PA-VSD, however, the current understanding of the role of CNVs in the etiology of PA-VSD is limited. There is just one report of rare *de novo* CNVs in patients with pulmonary atresia by Xie et al. (2014); however, it did not separate PA-IVS from PA-VSD. Thus, to investigate the pathogenesis of PA-VSD, we collected genomic DNA samples from sixty patients with PA-VSD and 100 controls; meanwhile, the samples of PA-IVS and TOF were also collected as positive control. All cases and controls were assayed using WES. Rare CNVs were identified in 100 patients and six of these CNVs were considered pathogenic or potentially pathogenic to PA-VSD.

Pulmonary atresia with ventricular septal defect is considered as the most severe type of TOF, and we intent to discover the different genomic causes of severity; whilst the genetic developmental pattern of PA-VSD partly differs from that of PA-IVS. Therefore, we compared rare CNVs between PA-VSD cases and positive controls (PA-IVS and TOF) to find the unique CNVs in PA-VSD. One CNV was a 344.5 kb deletion on 16p11.2 that contained PPP4C and TBX6. PPP4C, a catalytic subunit of protein phosphatase 4 which plays in various cellular signaling and regulation, is highly conserved from invertebrates to vertebrates (Cohen et al., 2005). Knockdown of ppp4c inhibits ventral development in zebrafish embryos via enhancing BMP signaling responses through its direct interaction with Smad1. Meanwhile, PPP4C also enhances BMP2 cellular responses in mammalian cells including mouse C2C12 myoblast cells (Jia et al., 2012). We all know that Bmp2-null mice show abnormal cardiac formation and BMP2 plays a pivotal role in cardiac development in human (Zhang and Bradley, 1996; Tan et al., 2017). It indicated that PPP4C could be implicated in human PA-VSD. The second gene, TBX6, was found in the same deletion as PPP4C. TBX6 is a member of the T-box family of transcription factors which are critical for normal heart development (Plageman and Yutzey, 2005). T-box genes are deemed to be important in early cardiac lineage determination and valvuloseptal development, including TBX1 (Yagi et al., 2003), TBX5 (Li et al., 1997), TBX20 (Plageman and Yutzey, 2004), and so on. Previous studies have revealed that Tbx6 has important roles in the formation of somite borders and the specification of presomitic mesoderm (Chapman et al., 1996; Chapman and Papaioannou, 1998; Chapman et al., 2003; White et al., 2003). Moreover, a recent research further indicated that Tbx6 was essential for pluripotent stem cells (PSCs) differentiation into mesoderm and inhibits cardiac specification (Sadahiro et al., 2018). The deletion CNV with TBX6 in our study may loss its function and result in the heart defects.

In addition, the 5q35.3 deletion contains the FLT4 gene, which encodes a receptor tyrosine kinase for VEGF-C and VEGF-D and promotes lymph angiogenesis as well as angiogenesis (Alitalo, 2011; Benedito et al., 2012). Moreover, FLT4 is highly expressed in the pulmonary arterial endothelial cells and interacts closely with BMPR2 to regulate BMP signaling which has an intimate association with cardiac development; genetic deletion of Flt4 in endothelial cells led to impaired BMP signaling in mouse (Hwangbo et al., 2017). It supports that the role of FLT4 in the pathogeny of PA-VSD is crucial.

The 5p13.1 deletion includes another interesting candidate gene RICTOR. RICTOR protein is an essential regulatory protein and structural subunit of the mammalian target of rapamycin complex 2 (mTORC2), which is a signaling protein complex involved in the epithelial-mesenchymal transition of embryonic development (Lamouille et al., 2012). Loss of endothelial homozygous Rictor results in mouse embryonic lethality at E11.5 (Aimi et al., 2015). It is reported that Rictor/mTORC2 may play a key role in the cardiomyocyte differentiation of the mouse embryonic stem cells with reduced protein levels of Nkx2.5 (cardiac progenitor cell protein), α-Actinin (cardiomyocyte biomarker), and brachyury (mesoderm protein) in Rictor knockdown mice during cardiogenesis. Furthermore, Rictor knockdown specifically suppressed the ventricular-like cells differentiation of the mouse embryonic stem cells (Zheng et al., 2017). These demonstrated that the crucial functions of RICTOR in heart development and potential pathogenesis of PA-VSD.

To a certain extent, PA-VSD and TOF show similar phenotype and may share the similar genetic mechanism. We identified a 6p21.33 duplication in both PA-VSD and TOF overlapping the TNXB gene, which encodes the tenascin-X protein. Tenascin is one of tendon-related extracellular matrix components (Peacock et al., 2008), expressing at the valve leaflets in chicken and mouse embryos as well as playing an important role in heart valve development (Lincoln et al., 2004; Combs and Yutzey, 2009).

Furthermore, PA-VSD shows the similar phenotype “pulmonary atresia” with PA-IVS, we intent to explain the similarity in genetic level by comparing the rare CNVs between PA-IVS and PA-VSD. For the common rare CNVs in PA-VSD and PA-IVS, 7p15.2 deletion was detected in this study and comprised HNRNPA2B1 gene. The HNRNPA2B1 gene, a molecular homolog of HNRNPA1 (Hutchison et al., 2002), has the similar structure and function to that of HNRNPA1 (Biamonti et al., 1994; Patry et al., 2003). The HNRNPA1 gene codes heterogeneous ribonucleoprotein (hnRNP) A1 protein, which is well-known trans-acting splicing factors that inhibit splice site recognition (Matlin et al., 2005) and promotes alternative splicing of target genes (Jean-Philippe et al., 2013). A recent study observed that the hnRNP A1 knockout mice had heart structure defects and the alternative splicing of mef2c was evidently affected (Liu et al., 2017). In our results, HNRNPA2B1 was highly expressed in human embryonic heart. Therefore, we inferred that HNRNPA2B1 may also play a role in heart development, especially involved in the formation of PA.

FGF22, another candidate gene within a duplication CNV at 19p13.3 locus, is most closely related to FGF7 and FGF10; these three FGFs constitute a subfamily among FGF family members (Miki et al., 1992; Ornitz et al., 1996; Yeh et al., 2003). Previous studies revealed that Fgf10 have dosage sensitive requirements in multiple aspects of early murine cardiovascular development (Urness et al., 2011) and plays an essential role in outflow tract morphogenesis (Kelly et al., 2001; Golzio et al., 2012). Although FGF22 was reported most in the neurology (Pasaoglu and Schikorski, 2016; Terauchi et al., 2016; Williams et al., 2016), we detected its variant in our patient populations with heart defects and speculated that FGF22 may have some similar function with FGF10 in heart development. Additionally, from the network we found that FGF22 directly interact with PDGFRA and KDR which are cardiac progenitor populations with Flk1 in differentiating embryonic stem cells (Kattman et al., 2006; Yang et al., 2008; Kattman et al., 2011). The result implied that FGF22 indeed have relation with heart development.

For the candidate genes, PPP4C, FLT4, RICTOR, and FGF22 showed strong interaction with all gene groups in networks analysis; meanwhile, RICTOR and PPP4C had high expression levels in human embryonic heart. It gives evidences that the rare CNVs of RICTOR and PPP4C contribute to pathogenesis of PA-VSD with great potential.

In conclusion, we identified three rare CNVs only in patients with PA-VSD and the putative candidate genes: 16p11.2 del (PPP4C), 5q35.3 del (FLT4) and 5p13.1 del (RICTOR). These rare CNVs and genes were not previously described and may contribute significantly to the genetic basis of PA-VSD. There were, however, limitations to this study. Our cohorts lacking parental samples and large or multicentric studies with trio samples may be needed for further replication studies to define the significance of the novel rare CNVs identified in our study. In order to minimize false positives in our small cohorts, the restricted CNV analytic methods were used for rare CNVs and it might have resulted in missing some rare variants of interest. Additionally, further mechanism studies are needed to prove the functional significance of putative candidate genes of PA-VSD *in vivo* or *in vitro*. Nevertheless, the discovery in our study of rare novel CNVs in patients with PA-VSD helps elucidate critical genes for PA-VSD and may provide new insights into understanding the pathogenesis of PA-VSD.

## Author Contributions

YY, KS, and HX contributed to conception and design of the study and performed the statistical analysis. NH, EZ, and FL collected the blood samples from all subjects. HX wrote the first draft of the manuscript. YY and KS revised the manuscript. All authors contributed to manuscript revision, read and approved the submitted version.

## Conflict of Interest Statement

The authors declare that the research was conducted in the absence of any commercial or financial relationships that could be construed as a potential conflict of interest.

## Footnotes

1 http://broadinstitute.github.io/picard/

2 https://software.broadinstitute.org/gatk/

3 http://dgv.tcag.ca/

4 http://www.malacards.org/
