# Supplementary material for: Identification of Rare Copy Number Variants Associated With Pulmonary Atresia With Ventricular Septal Defect
Source: Front Genet. 2019 Jan 28;10:15. doi: 10.3389/fgene.2019.00015 (PMC6360179; doi:10.3389/fgene.2019.00015)
Supplement: Supplementary file 1 [file Table_1.DOCX]

Supplementary Material

Identification of rare copy number variants associated with pulmonary atresia with ventricular septal defect

Huilin Xie^1^, Nanchao Hong^1^, Erge Zhang^1^, Fen Li^2^, Kun Sun^1*^, Yu Yu^1*^

*** Correspondence:**Kun Sun
[sunkun@xinhuamed.com.cn](mailto:sunkun@xinhuamed.com.cn)
Yu Yu
[yuyu@xinhuamed.com.cn](mailto:yuyu@xinhuamed.com.cn)

# Supplementary Tables

**Supplementary Table S1:** Known gene lists from previous studies and Malacards database for network analysis.

| List 1 | | | List 2 | | | List 3 | | |
| --- | --- | --- | --- | --- | --- | --- | --- | --- |
| Gene | Relevance | Source | Gene | Relevance | Source | Gene | Relevance | Source |
| GATA4^1^ | PS、ASD | PubMed | KDR^31^ | Vessel | PubMed | SPRED1^36^ | LS | PubMed |
| MYH7^2^ | PS、ASD | PubMed | MESP1^32^ | Vessel | PubMed | MAP2K1^37^ | LS, NS | PubMed |
| GATA6^3^ | PS | PubMed | Hif-1a^33^ | Vessel | PubMed | KRAS^37^ | LS, NS | PubMed |
| NOS3^4^ | PS | PubMed | Egr1^34^ | VSMC | PubMed | NF1^36^ | LS | PubMed |
| Postn^5^ | PS | PubMed | F3^35^ | VSMC | PubMed | PTPN11^38^ | LS, NS | PubMed |
| TGFβ^5^ | PS | PubMed | Ptgs2^35^ | VSMC | PubMed | RAF1^36^ | LS, NS | PubMed |
| Lox^5^ | PS | PubMed | Serpine1^35^ | VSMC | PubMed | BRAF^37^ | LS, NS | PubMed |
| Mfap4^5^ | PS | PubMed | Lypd8^35^ | VSMC | PubMed | FER^39^ | LS, NS | PubMed |
| Sfrp2^5^ | PS | PubMed | Cx3cl1^35^ | VSMC | PubMed | MPZL1^40^ | LS, NS | PubMed |
| DKK3^5^ | PS | PubMed | Cav1^35^ | VSMC | PubMed | Epha2^41^ | LS | PubMed |
| Wif1^5^ | PS | PubMed | Rhoa^35^ | VSMC | PubMed | NRAS^36^ | NS | PubMed |
| DHFR^6^ | PA | PubMed | Nppb^35^ | VSMC | PubMed | RRAS^42^ | NS | PubMed |
| CUBN^6^ | PA | PubMed | Prrx1^35^ | VSMC | PubMed | SOS1^36^ | NS, LS | PubMed |
| CAMTA2^6^ | PA | PubMed | ANGPT2^7^ | Vessel | PubMed | SOS2^43^ | NS | PubMed |
| PDE8B^6^ | PA | PubMed | FGF10^7^ | Vessel | PubMed | CBL^36^ | NS | PubMed |
| AP3B1^6^ | PA | PubMed |  |  |  | RASA2^44^ | NS | PubMed |
| ARSB^6^ | PA | PubMed |  |  |  | SHOC2^36^ | NS, LS | PubMed |
| DMGDH^6^ | PA | PubMed |  |  |  | MEK1^37^ | NS, LS | PubMed |
| MYH11^6^ | PA | PubMed |  |  |  | RIT1^44^ | NS, LS | PubMed |
| MTHFR^6^ | PA | PubMed |  |  |  | HRAS^37^ | NS, LS | PubMed |
| PLXNA2^7^ | TOF | PubMed |  |  |  | JAG1^13^ | AS | PubMed |
| GJA5^8^ | TOF、OFT | PubMed |  |  |  | CTP | CS | PubMed |
| EHMT1^9^ | TOF | PubMed |  |  |  | DVL22 | CS | PubMed |
| GLRX2^10^ | Heart | PubMed |  |  |  | CFC1 | CS | PubMed |
| NFATC1^11^ | Valve | PubMed |  |  |  | ARVCF | VCFS | PubMed |
| TBX1^12^ | CHD | PubMed |  |  |  | GP1BB | VCFS | Malacards |
| JAG1^13^ | CHD | PubMed |  |  |  | COMT | VCFS | Malacards |
| ZFPM2^14^ | CHD | PubMed |  |  |  | UFD1 | VCFS | Malacards |
| NKX2.5^15^ | CHD | PubMed |  |  |  | HIRA | VCFS, DGS | Malacards |
| Sema3c^16^ | OFT | PubMed |  |  |  | JMJD1C | VCFS | Malacards |
| Foxc1^16^ | OFT、SHF | PubMed |  |  |  | SEC24C | VCFS | Malacards |
| Foxc2^16^ | OFT、SHF | PubMed |  |  |  | DGCR6 | VCFS, DGS | Malacards |
| Pdgfrα^17^ | OFT | PubMed |  |  |  | RREB1 | VCFS | Malacards |
| SOX4^18^ | OFT | PubMed |  |  |  | TBX1 | DGS | Malacards |
| SOX11^18^ | OFT | PubMed |  |  |  | DGCR14 | DGS | Malacards |
| Isl1^19^ | SHF | PubMed |  |  |  | DGCR2 | DGS | Malacards |
| Six2^20^ | SHF | PubMed |  |  |  | DGCR8 | DGS | Malacards |
| MASP1^21^ | CNCC | PubMed |  |  |  | DGCR6L | DGS | Malacards |
| Meis2^22^ | CNCC | PubMed |  |  |  | GNB1L | DGS | Malacards |
| NUMB^23^ | CNCC | PubMed |  |  |  | LZTR1 | DGS | Malacards |
| NUNML | CNCC | PubMed |  |  |  | ACF | CCS | Malacards |
| Ets1^24^ | CNCC | PubMed |  |  |  | TBX5^45^ | HOS | Malacards |
| Sox9^25^ | NCC | PubMed |  |  |  | TFAP2b^46^ | Char S | PubMed |
| Sox10^25^ | NCC | PubMed |  |  |  | CHD7^47^ | Charge S | PubMed |
| FoxD3^24^ | NCC | PubMed |  |  |  | EVC^48^ | ECS | PubMed |
| Snai2^25^ | NCC | PubMed |  |  |  | FBN1^49^ | MFS | PubMed |
| Msx1^24^ | NCC | PubMed |  |  |  |  |  |  |
| Pax3^24^ | NCC | PubMed |  |  |  |  |  |  |
| Pax7^24^ | NCC | PubMed |  |  |  |  |  |  |
| Zic1^24^ | NCC | PubMed |  |  |  |  |  |  |
| Meis1^26^ | NCC | PubMed |  |  |  |  |  |  |
| Pax1^27^ | NCC | PubMed |  |  |  |  |  |  |
| FGF8^28^ | CHD | PubMed |  |  |  |  |  |  |
| BMP2^29^ | CHD | PubMed |  |  |  |  |  |  |
| BMP4^29^ | CHD | PubMed |  |  |  |  |  |  |
| WNT1^30^ | CHD | PubMed |  |  |  |  |  |  |
| WNT5a^30^ | CHD | PubMed |  |  |  |  |  |  |

PS, pulmonary stenosis; ASD, atrial septal defect; PA, pulmonary atresia; TOF, tetralogy of Fallot; OFT, outflow tract; Heart, heart development; CHD, congenital heart disease; Valve, valvular development; SHF, secondary heart field; CNCC, cardiac neural crest cell; NCC, neural crest cell; Vessel, blood vessel development; VSMC, vascular smooth muscle cell proliferation; LS, LEOPARD syndrome; NS, Noonan syndrome; AS, Alagille syndrome; CS, CATCH22 syndrome; VCFS, Velo-Cardio-Facial syndrome; DGS, DiGeorge syndrome; CCS, Cayler Cardiofacial syndrome; HOS, Holt-Oram syndrome; Char S, Char syndrome; Charge S, Charge syndrome; ECS, Ellis-van Creveld syndrome; MFS, Marfan syndrome.

**References**

1. Garg, V., Kathiriya, I.S., Barnes, R., Schluterman, M.K., King, I.N., Butler, C.A., et al. (2003). GATA4 mutations cause human congenital heart defects and reveal an interaction with TBX5. Nature. 424, 443-447.

2. Wang, W., Niu, Z., Wang, Y., Li, Y., Zou, H., Yang, L., et al. (2016). Comparative transcriptome analysis of atrial septal defect identifies dysregulated genes during heart septum morphogenesis. Gene. 575, 303-312. doi: 10.1016/j.gene.2015.09.016.

3. Catli, G., Abaci, A., Flanagan, S.E., De Franco, E., Ellard, S., Hattersley, A., et al. (2013). A novel GATA6 mutation leading to congenital heart defects and permanent neonatal diabetes: a case report. Diabetes Metab. 39, 370-374. doi: 10.1016/j.diabet.2013.01.005.

4. Kuehl, K., Loffredo, C., Lammer, E.J., Iovannisci, D.M., Shaw, G.M. (2010). Association of congenital cardiovascular malformations with 33 single nucleotide polymorphisms of selected cardiovascular disease-related genes. Birth Defects Res A Clin Mol Teratol. 88, 101-110. doi: 10.1002/bdra.20630.

5. Urashima, T., Zhao, M., Wagner, R., Fajardo, G., Farahani, S., Quertermous, T., et al. (2008). Molecular and physiological characterization of RV remodeling in a murine model of pulmonary stenosis. Am J Physiol Heart Circ Physiol. 295, H1351-H1368. doi: 10.1152/ajpheart.91526.2007.

6. Xie, L., Chen, J.L., Zhang, W.Z., Wang, S.Z., Zhao, T.L., Huang, C., et al. (2014). Rare de novo copy number variants in patients with congenital pulmonary atresia. PLoS One. 9, e96471. doi: 10.1371/journal.pone.0096471.

7. Silversides, C.K., Lionel, A.C., Costain, G., Merico, D., Migita, O., Liu, B., et al. (2012). Rare copy number variations in adults with tetralogy of Fallot implicate novel risk gene pathways. PLoS Genet. 8, e1002843. doi: 10.1371/journal.pgen.1002843.

8. Guida, V., Ferese, R., Rocchetti, M., Bonetti, M., Sarkozy, A., Cecchetti, S., et al. (2013). A variant in the carboxyl-terminus of connexin 40 alters GAP junctions and increases risk for tetralogy of Fallot. Eur J Hum Genet. 21, 69-75. doi: 10.1038/ejhg.2012.109.

9. Tansatit, M., Kongruttanachok, N., Kongnak, W., Arunpan, S., Maneeshote, P., Buasorn, V., et al. (2006). Tetralogy of Fallot with absent pulmonary valve in a de novo derivative chromosome 9 with duplication of 9p13 --> 9pter and deletion of 9q34.3. Am J Med Genet A. 140, 1981-1987.

10. Mailloux, R.J., Xuan, J.Y., McBride, S., Maharsy, W., Thorn, S., Holterman, C.E., et al. (2014). Glutaredoxin-2 is required to control oxidative phosphorylation in cardiac muscle by mediating deglutathionylation reactions. J Biol Chem. 289, 14812-14828. doi: 10.1074/jbc.M114.550574.

11. Yehya, A., Souki, R., Bitar, F., Nemer, G. (2006). Differential duplication of an intronic region in the NFATC1 gene in patients with congenital heart disease. Genome. 49, 1092-1098.

12. Yagi, H., Furutani, Y., Hamada, H., Sasaki, T., Asakawa, S., Minoshima, S., et al. (2003). Role of TBX1 in human del22q11.2 syndrome. Lancet. 362, 1366-1373.

13. Oda, T., Elkahloun, A.G., Pike, B.L., Okajima, K., Krantz, I.D., Genin, A., et al. (1997). Mutations in the human Jagged1 gene are responsible for Alagille syndrome. Nat Genet. 16, 235-242.

14. Sarkozy, A., Conti, E., D'Agostino, R., Digilio, M.C., Formigari, R., Picchio, F., et al. (2005). ZFPM2/FOG2 and HEY2 genes analysis in nonsyndromic tricuspid atresia. Am J Med Genet A. 133A, 68-70.

15. Schott, J.J., Benson, D.W., Basson, C.T., Pease, W., Silberbach, G.M., Moak, J.P., et al. (1998). Congenital heart disease caused by mutations in the transcription factor NKX2-5. Science. 281, 108-111.

16. Kodo, K., Shibata, S., Miyagawa-Tomita, S., Ong, S.G., Takahashi, H., Kume, T., et al. (2017). Regulation of Sema3c and the Interaction between Cardiac Neural Crest and Second Heart Field during Outflow Tract Development. Sci Rep. 7, 6771. doi: 10.1038/s41598-017-06964-9.

17. Aghajanian, H., Cho, Y.K., Rizer, N.W., Wang, Q., Li, L., Degenhardt, K., et al. (2017). Pdgfrα functions in endothelial-derived cells to regulate neural crest cells and the development of the great arteries. Dis Model Mech. 10, 1101-1108. doi: 10.1242/dmm.029710.

18. Paul, M.H., Harvey, R.P., Wegner, M., Sock, E. (2014). Cardiac outflow tract development relies on the complex function of Sox4 and Sox11 in multiple cell types. Cell Mol Life Sci. 71, 2931-2945. doi: 10.1007/s00018-013-1523-x.

19. Snarr, B.S., O'Neal, J.L., Chintalapudi, M.R., Wirrig, E.E., Phelps, A.L., Kubalak, S.W., et al. (2007). Isl1 expression at the venous pole identifies a novel role for the second heart field in cardiac development. Circ Res. 101, 971-974.

20. Zhou, Z., Wang, J., Guo, C., Chang, W., Zhuang, J., Zhu, P., et al. (2017). Temporally Distinct Six2-Positive Second Heart Field Progenitors Regulate Mammalian Heart Development and Disease. Cell Rep. 18, 1019-1032. doi: 10.1016/j.celrep.2017.01.002.

21. Rooryck, C., Diaz-Font, A., Osborn, D.P., Chabchoub, E., Hernandez-Hernandez, V., Shamseldin, H., et al. (2011). Mutations in lectin complement pathway genes COLEC11 and MASP1 cause 3MC syndrome. Nat Genet. 43, 197-203. doi: 10.1038/ng.757.

22. Machon, O., Masek, J., Machonova, O., Krauss, S., Kozmik, Z. (2015). Meis2 is essential for cranial and cardiac neural crest development. BMC Dev Biol. 15, 40. doi: 10.1186/s12861-015-0093-6.

23. Zhao, C., Guo, H., Li, J., Myint, T., Pittman, W., Yang, L., et al. (2014). Numb family proteins are essential for cardiac morphogenesis and progenitor differentiation. Development. 141, 281-295. doi: 10.1242/dev.093690.

24. Simões-Costa, M.S., McKeown, S.J., Tan-Cabugao, J., Sauka-Spengler, T., Bronner, M.E. (2012). Dynamic and differential regulation of stem cell factor FoxD3 in the neural crest is Encrypted in the genome. PLoS Genet. 8, e1003142. doi: 10.1371/journal.pgen.1003142.

25. Liu, J.A., Wu, M.H., Yan, C.H., Chau, B.K., So, H., Ng, A., et al. (2013). Phosphorylation of Sox9 is required for neural crest delamination and is regulated downstream of BMP and canonical Wnt signaling. Proc Natl Acad Sci U S A. 110, 2882-2887. doi: 10.1073/pnas.1211747110.

26. Maeda, R., Mood, K., Jones, T.L., Aruga, J., Buchberg, A.M., Daar, I.O. (2001). Xmeis1, a protooncogene involved in specifying neural crest cell fate in Xenopus embryos. Oncogene. 20, 1329-1342.

27. Chuang, S.F., Su, L.H., Cho, C.C., Pan, Y.J., Sun, C.H. (2012). Functional redundancy of two Pax-like proteins in transcriptional activation of cyst wall protein genes in Giardia lamblia. PLoS One. 7, e30614. doi: 10.1371/journal.pone.0030614.

28. Ilagan, R., Abu-Issa, R., Brown, D., Yang, Y.P., Jiao, K., Schwartz, R.J., et al. (2006). Fgf8 is required for anterior heart field development. Development. 133, 2435-2445.

29. Stottmann, R.W., Choi, M., Mishina, Y., Meyers, E.N., Klingensmith, J. (2004). BMP receptor IA is required in mammalian neural crest cells for development of the cardiac outflow tract and ventricular myocardium. Development. 131, 2205-2218.

30. Li, D., Sinha, T., Ajima, R., Seo, H.S., Yamaguchi, T.P., Wang, J. (2016). Spatial regulation of cell cohesion by Wnt5a during second heart field progenitor deployment. Dev Biol. 412, 18-31. doi: 10.1016/j.ydbio.2016.02.017.

31. Ishitobi, H., Matsumoto, K., Azami, T., Itoh, F., Itoh, S., Takahashi, S., et al. (2010). Flk1-GFP BAC Tg mice: an animal model for the study of blood vessel development. Exp Anim. 59, 615-622.

32. David, R., Brenner, C., Stieber, J., Schwarz, F., Brunner, S., Vollmer, M., et al. (2008). MesP1 drives vertebrate cardiovascular differentiation through Dkk-1-mediated blockade of Wnt-signalling. Nat Cell Biol. 10, 338-345. doi: 10.1038/ncb1696.

33. Gerri, C., Marín-Juez, R., Marass, M., Marks, A., Maischein, H.M., Stainier, D.Y.R. (2017). Hif-1α regulates macrophage-endothelial interactions during blood vessel development in zebrafish. Nat Commun. 8, 15492. doi: 10.1038/ncomms15492.

34. Han, W., Liu, G.N. (2010). EGR-1 decoy ODNs inhibit vascular smooth muscle cell proliferation and neointimal hyperplasia of balloon-injured arteries in rat. Life Sci. 86, 234-243. doi: 10.1016/j.lfs.2009.12.005.

35. Huhtinen A, Hongisto V, Laiho A, Löyttyniemi E, Pijnenburg D, Scheinin M. (2017). Gene expression profiles and signaling mechanisms in α2B-adrenoceptor-evoked proliferation of vascular smooth muscle cells. BMC Syst Biol. 11, 65. doi: 10.1186/s12918-017-0439-8.

36. Tartaglia, M., Gelb, B.D., Zenker, M. (2011). Noonan syndrome and clinically related disorders. Best Pract Res Clin Endocrinol Metab. 25, 161-179. doi: 10.1016/j.beem.2010.09.002.

37. Aoki, Y., Niihori, T., Narumi, Y., Kure, S., Matsubara, Y. (2008). The RAS/MAPK syndromes: novel roles of the RAS pathway in human genetic disorders. Hum Mutat. 29, 992-1006. doi: 10.1002/humu.20748.

38. Araki, T., Mohi, M.G., Ismat, F.A., Bronson, R.T., Williams, I.R., Kutok, J.L., et al. (2004). Mouse model of Noonan syndrome reveals cell type- and gene dosage-dependent effects of Ptpn11 mutation. Nat Med. 10, 849-857.

39. Paardekooper Overman, J., Preisinger, C., Prummel, K., Bonetti, M., Giansanti, P., Heck, A., et al. (2014). Phosphoproteomics-mediated identification of Fer kinase as a target of mutant Shp2 in Noonan and LEOPARD syndrome. PLoS One. 9, e106682. doi: 10.1371/journal.pone.0106682.

40. Paardekooper Overman, J., Yi, J.S., Bonetti, M., Soulsby, M., Preisinger, C., Stokes, M.P., et al. (2014). PZR coordinates Shp2 Noonan and LEOPARD syndrome signaling in zebrafish and mice. Mol Cell Biol. 34, 2874-2889. doi: 10.1128/MCB.00135-14.

41. Miura, K., Wakayama, Y., Tanino, M., Orba, Y., Sawa, H., Hatakeyama, M., et al. (2013). Involvement of EphA2-mediated tyrosine phosphorylation of Shp2 in Shp2-regulated activation of extracellular signal-regulated kinase. Oncogene. 32, 5292-5301. doi: 10.1038/onc.2012.571.

42. Flex, E., Jaiswal, M., Pantaleoni, F., Martinelli, S., Strullu, M., Fansa, E.K., et al. (2014). Activating mutations in RRAS underlie a phenotype within the RASopathy spectrum and contribute to leukaemogenesis. Hum Mol Genet. 23, 4315-4327. doi: 10.1093/hmg/ddu148.

43. Cordeddu, V., Yin, J.C., Gunnarsson, C., Virtanen, C., Drunat, S., Lepri, F., et al. (2015). Activating Mutations Affecting the Dbl Homology Domain of SOS2 Cause Noonan Syndrome. Hum Mutat. 36, 1080-1087. doi: 10.1002/humu.22834.

44. Chen, P.C., Yin, J., Yu, H.W., Yuan, T., Fernandez, M., Yung, C.K., et al. (2014). Next-generation sequencing identifies rare variants associated with Noonan syndrome. Proc Natl Acad Sci U S A. 111, 11473-11478. doi: 10.1073/pnas.1324128111.

45. Basson, C.T., Bachinsky, D.R., Lin, R.C., Levi, T., Elkins, J.A., Soults, J., et al. (1997). Mutations in human TBX5 cause limb and cardiac malformation in Holt-Oram syndrome. Nat Genet. 15, 30-35.

46. Satoda, M., Zhao, F., Diaz, G.A., Burn, J., Goodship, J., Davidson, H.R., et al. (2000). Mutations in TFAP2B cause Char syndrome, a familial form of patent ductus arteriosus. Nat Genet. 25, 42-46.

47. Okuno, H., Renault Mihara, F., Ohta, S., Fukuda, K., Kurosawa, K., Akamatsu, W., et al. (2017). CHARGE syndrome modeling using patient-iPSCs reveals defective migration of neural crest cells harboring CHD7 mutations. Elife. 6. Pii, e21114. doi: 10.7554/eLife.21114.

48. D'Asdia, M.C., Torrente, I., Consoli, F., Ferese, R., Magliozzi, M., Bernardini, L., et al. (2013). Novel and recurrent EVC and EVC2 mutations in Ellis-van Creveld syndrome and Weyers acrofacial dyostosis. Eur J Med Genet. 56, 80-87. doi: 10.1016/j.ejmg.2012.11.005.

49. Mellody, K.T., Freeman, L.J., Baldock, C., Jowitt, T.A., Siegler, V., Raynal, B.D., et al. (2006). Marfan syndrome-causing mutations in fibrillin-1 result in gross morphological alterations and highlight the structural importance of the second hybrid domain. J Biol Chem. 281, 31854-31862.
